# Fertility Intentions and Use of Contraception among Monogamous Couples in Northern Malawi in the Context of HIV Testing: A Cross-Sectional Analysis

**DOI:** 10.1371/journal.pone.0051861

**Published:** 2012-12-20

**Authors:** Albert L. N. Dube, Angela Baschieri, John Cleland, Sian Floyd, Anna Molesworth, Fiona Parrott, Neil French, Judith R. Glynn

**Affiliations:** 1 Karonga Prevention Study, London School of Hygiene and Tropical Medicine, Karonga, Malawi; 2 Department of Population Health, London School of Hygiene, and Tropical Medicine, London, United Kingdom; 3 Department of Infectious Disease Epidemiology, London School of Hygiene and Tropical Medicine, London, United Kingdom; 4 Institute of Infection and Global Health, University of Liverpool, Liverpool, United Kingdom; University of Ottawa, Canada

## Abstract

**Context:**

Knowledge of HIV status may influence fertility desires of married men and women. There is little knowledge about the importance of this influence among monogamously married couples and how knowledge of HIV status influences use of contraception among these couples.

**Methodology:**

We carried out a cross-sectional analysis of interview data collected between October 2008 and September 2009 on men aged 15–59 years and women aged 15–49 years who formed 1766 monogamously married couples within the Karonga Prevention Study demographic surveillance study in northern Malawi.

**Results:**

5% of men and 4% of women knew that they were HIV positive at the time of interview and 81% of men and 89% of women knew that they were HIV negative. 73% of men and 83% of women who knew that they were HIV positive stated that they did not want more children, compared to 35% of men and 38% of women who knew they were HIV negative. Concordant HIV positive couples were more likely than concordant negative couples to desire to stop child bearing (odds ratio 11.5, 95%CI 4.3–30.7, after adjusting for other factors) but only slightly more likely to use contraceptives (adjusted odds ratio 1.5 (95%CI 0.8–3.3).

**Conclusion:**

Knowledge of HIV positive status is associated with an increase in the reported desire to cease childbearing but there was limited evidence that this desire led to higher use of contraception. More efforts directed towards assisting HIV positive couples to access and use reproductive health services and limit HIV transmission among couples are recommended.

## Introduction

Knowledge of HIV status could influence desired fertility in two opposing ways [1]. Knowledge of HIV positive status could reduce the desire for additional children through concern for the health consequences for the mother and child and ability to care for the children until maturity. Alternatively, awareness of HIV status could accelerate childbearing for fear of not reaching a desired family size before the onset of AIDS or due to an increased desire for more children because of high levels of child mortality [2], [3], [4].

In the mid 1990s testing for HIV was not widespread and an HIV diagnosis late in the course of disease implied that many infected people could not survive long enough to change their fertility [3]. As a consequence, studies on the effect of HIV on fertility found that although being HIV positive is associated with lower fertility, knowledge of HIV positive status did not translate into a noticeable negative or positive impact on fertility [1], [3].

Research to date on knowledge of HIV status and couples’ fertility intentions has been limited. Individual level studies reveal that HIV positive individuals want to limit childbearing [5], [6], [7] in consideration of factors such as risk of infection to their partners and their unborn children [8]; awareness that the onset of AIDS will lead to ill-health and increased difficulties in caring for themselves and their children [9]; perceived community disapproval associated with HIV and reproduction even though motherhood and childbearing are socially valuable for young HIV positive people [10]. These factors have varied by gender with women expressing more concern about the health consequences of carrying a pregnancy to full-term and childbearing while men are more concerned about their own early death and the future of their children [11]. Men and women who tested positive and yet had perceived themselves as being HIV negative changed their fertility intentions from desiring to have more children to desiring to stop [12].

While research findings have been consistent on the effect of HIV awareness on fertility intentions, studies of the effect of antiretroviral treatment (ART) on fertility intentions have tended to be inconclusive [8], [13], [14]. Studies in Uganda and South Africa found that women on ART were more likely to want more children compared to women who were not on ART and that this desire increased with duration of being on ART [13], [14] But other studies found no clear or no evidence that being enrolled in an ART treatment clinic modified fertility intentions [5], [9].

Few studies have analysed the relationship between fertility intentions and HIV awareness in *couples* and how spousal differences in fertility intentions influence use of contraception. A review of these studies found mixed and inconsistent evidence of which partner carries more weight in reproductive decisions [15]. Another review argued that couple’s joint fertility intentions lead to better predictions of subsequent behaviour [16]. Doodo [17] analysing two rounds of demographic and health survey data from Ghana and Kenya compared reproductive intentions of men and women; he found little evidence that men’s preferences are more influential. A comparative study in 18 countries using couples’ data from the Demographic and Health Survey found that there is a process of negotiation between couples with different reproductive goals and that use of modern methods of contraception was higher when both want to stop, except in Malawi where use was higher when the man, but not the wife, wanted to stop. [18].

This study, using data on fertility intentions and information on HIV status of couples linked to an on-going demographic surveillance system in Karonga district in Northern Malawi will investigate how knowledge of HIV status alters men’s, women’s and couples’ fertility intentions and how existing differences in fertility intentions influence use of contraception. We achieve this by answering the following specific research questions:

1. How does knowledge of HIV status influence fertility intentions for men, women and couples?
2. How does knowledge of HIV status influence contraceptive use?

## Methods

This study uses data collected between October 2008 and September 2009 from a module on fertility intentions linked to an on-going Demographic Surveillance system (DSS). The DSS baseline census was conducted in 2002–2004 in a population of around 33,000 individuals, following which the population has been under continuous surveillance with an annual re-census. A population-based adult HIV and behaviour survey started in the DSS area in September 2007 [19], [20].

Questions, asked separately to men and women, using the local language (Tumbuka), captured marital status, current fertility, including total number of children ever born and surviving (separating those born with the current spouse and those born from past unions), fertility intentions, including perception of their partner’s desire and the preferred timing of the next birth, and use of contraception. Respondents who wanted no more children were also asked consequences of having another child (as an open question). Unique individual identification numbers in the DSS permits the linking of couples’ intentions data. Interviews were conducted by trained interviewers, one-to-one and in private.

This paper analyses the baseline round of data (2008–2009) that is part of three rounds of data collection: 2008–2009, 2009–2010, and 2010–2011 approved by the National Health Research Ethic Committee of the National Commission for Science and Technology of Malawi. Data on fertility were collected along with data on sexual behaviour as part of a survey which also offered HIV testing. Individuals could refuse testing and still complete the questionnaire. The data were collected approximately one month after the re-census of that area, which included collection of socio-economic data and identification of spouses.

We collected data on HIV status between October 2007 and October 2008 using door-to-door HIV testing with rapid tests one year before the onset of the fertility intentions study [20]. We collected data on knowledge of HIV status and whether the participants have ever been enrolled on ART after filling fertility intentions questions. Free ART has been available in Karonga District since 2005 and in the DSS area since 2006. For the analysis we used stated knowledge of HIV status rather than actual status, since it is knowledge that will influence behaviour.

### Ethics

Ethics approval for the study was received from the Health Sciences Research Committee, Malawi, and the ethics committee of the London School of Hygiene & Tropical Medicine, UK. Before the start of the demographic surveillance the Traditional Authority that covers the area, and all village headmen and traditional advisors in the study area were informed about the aims of the study and the nature of the data to be collected, and their approval and verbal consent was sought. All household members were given a similar explanation and interviews were only conducted if verbal consent was given by the household head and by the respective household members. The consent for the demographic surveillance was recorded by the interview sheet being filled. Refusals were recorded in field registers. During the baseline census 15 households did not provide verbal consent and have consequently been excluded from the study. The socio-demographic data for this study come from the basic demographic surveillance for which the ethics committees agreed that written consent was not needed. For the fertility intentions survey and for HIV testing, separate individual written consent was sought.

### Statistical Analysis

We used Stata 11 software to analyse data. We carried out bi-variate analysis using Chi-square tests for associations and then ran logistic regression analyses for the probability of wanting no more children. We examined whether fertility intentions of couples varied by their HIV status. There were four possible outcome combinations for the couples: both wanting more children or undecided; both wanting no more children; wife wanting no more children husband wanting more/undecided; husband wanting no more children wife wanting more/undecided. The undecided were grouped with those who wanted more children because the undecided tend to be classified the same as the majority among those in the survey with stated desires [21]. Owing to small numbers, we subsequently collapsed this outcome variable to two categories: both want no more children and both or either want more or undecided. There were also four different ways in which knowledge of HIV status could be distributed in the couple: concordant positive; concordant negative; discordant husband positive, discordant wife positive. Using logistic regression we also explored how couples’ knowledge of their HIV status influences use of contraception. We used women’s reports of use of contraception to represent couples’ use of contraception.

## Results

**Figure 1:**
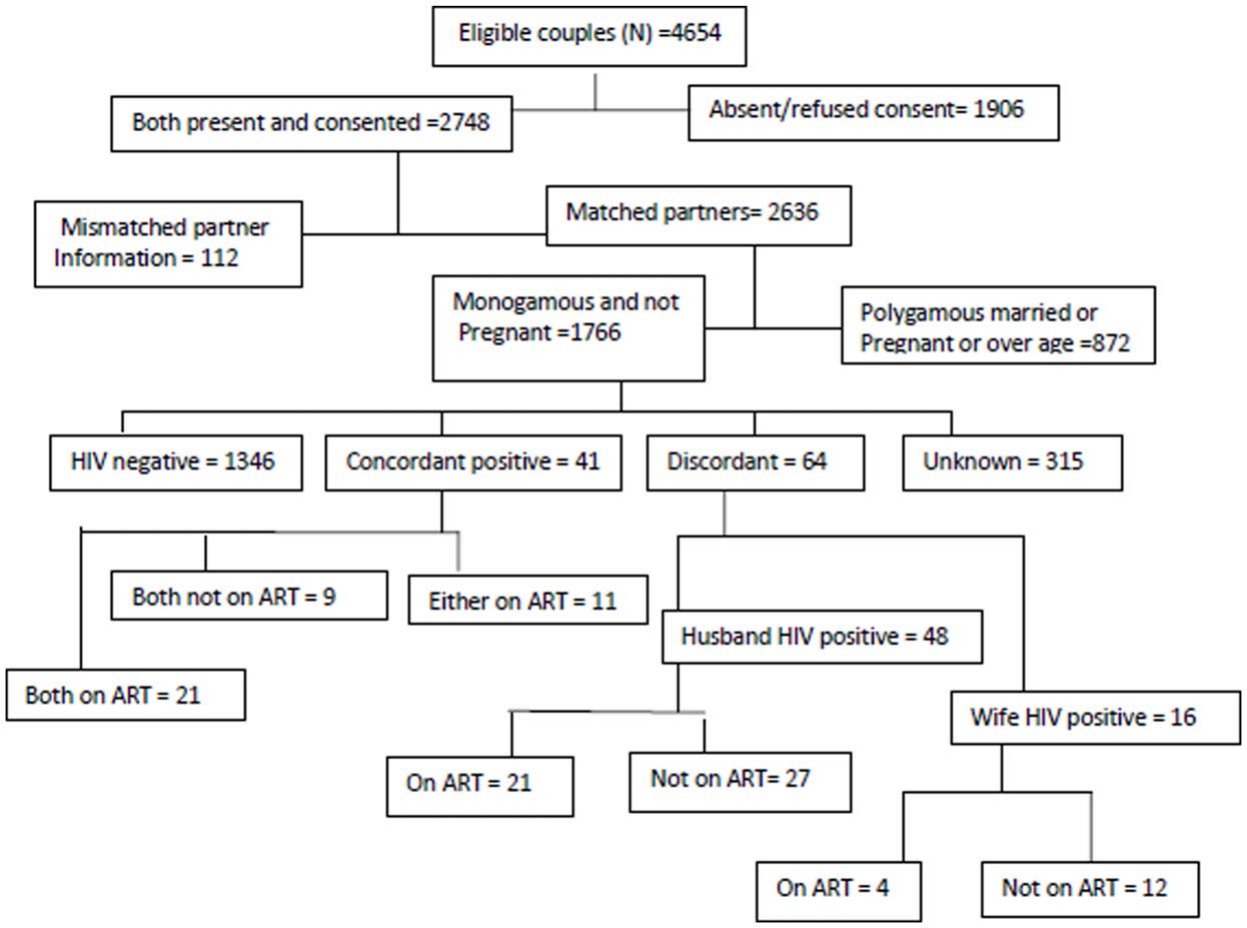
Monogamous married couples’ inclusion, HIV and ART status chart.

Of 4654 married couples known from the census, there were 2748 couples where both husband and wife were seen agreed to take part in the fertility intentions study. We dropped 112 records due to mismatching information on the partner’s identity, marital status or the name of the wife or husband. We obtained information on 2636 matched unions of which 67% were monogamous couples and not currently pregnant. We excluded 11%who were pregnant, 20% polygamous and 2% who were outside childbearing age group. Figure 1 presents information on how couples were included into the analysis, their Knowledge of HIV status and use of ART.

The mean age of women was 29 years (range 15–49 years) with HIV positive women being slightly older (mean age 33 years range 17 to 48) than HIV negative women (mean age 29 years, range 15 to 49). For the men the mean age was 35 years (range 17–66 years) with HIV positive men being older (mean age 43 years range 25 to 64) than HIV negative men (mean age 35 years range 17 to 66 ). The mean duration of marriage was 9.8 years with a range of 0 to 35 years. 92/1766 men (5%) knew that they were HIV positive and 66/1766 women (4%) knew that they were HIV positive. HIV testing at the time of the survey revealed that an additional 17 women and 7 men were HIV positive. Based on previous test results from sero-surveys and others studies carried out among the study population, 98% of those who reported being HIV negative were really HIV negative at the time they last had an HIV test before the survey (1139/1160 men and 1318/1341 women with results available). 100% (64/64) of those who reported being HIV positive were indeed HIV positive. 35/51 HIV positive women reported to have disclosed their HIV status to their husbands and 59/82 HIV positive men reported to have disclosed their HIV status to their wives. The proportions were similar for HIV negative men and women: 68% (866/1262) and 69% (890/1293) respectively had disclosed.

Table 1 shows the proportion of men and women wanting no more children, and proportion of women using contraception, by background characteristics of the study participants. The proportion wanting no more children increased with age, number of children and level of education among both men and women. We also found that the proportion wanting no more children increased with increasing duration of marriage. Fertility intentions differed by knowledge of HIV status. Both men and women who reported that they were HIV positive, either on ART or not on ART, were twice as likely to report a desire to stop child bearing compared to men and women who reported that they were HIV negative.

**Table 1:** Fertility intentions by background characteristics.

	Fertility intentions (N = 1766)							
Background characteristics	% wanting more children		% wanting no more children		% missing intention		Number	
	Men	Women	Men	Women	Men	Women	Men	Women
**Reported HIV Status**								
Negative	63	60	35	38	2	2	1437	1574
Positive on ART	29	14	70	86	1	0	56	29
Positive not on ART	28	24	69	73	3	3	36	37
Unknown	54	60	43	39	3	1	237	126
**Age**								
15–24	88	88	9	11	3	1	223	643
25–34	77	58	22	41	1	1	715	686
35–44	48	19	51	80	1	1	488	347
45 plus	25	3	70	87	5	10	340	90
**Number of living children**								
No child	68	70	32	20	0	10	60	154
1–3 children	77	77	22	23	1	0	877	962
4–6 children	43	32	54	68	3	0	544	525
7 plus	21	14	76	82	3	4	183	125
Missing	76	–	17	–	7	–	102	–
**Child Mortality**								
No child death	64	64	34	34	2	2	1373	1308
Child death	47	42	51	57	2	1	393	458
**Education**								
None/Primary 1–5 grade	64	54	34	44	2	2	412	782
Primary –6–8 grade	56	55	42	43	2	2	576	595
Lower Secondary	65	72	34	27	1	1	415	307
Upper Secondary/Tertiary	57	72	39	28	4	0	363	82
**Dwelling category**								
Richest	47	46	49	53	4	1	346	346
2	53	53	46	46	1	1	275	275
3	61	59	37	39	2	2	663	663
Poor	72	70	26	28	2	2	482	482

Using Fishers Exact Test we found no significant difference between those on ART and those not on ART and therefore combined these two groups.

Table 2 presents unadjusted and adjusted odds ratios (OR) predicting the likelihood of wanting no more children among married men and women. Adjustment for a wide range of possible confounders made little difference. We found that HIV positive individuals were more likely to want no more children compared to their HIV negative counter parts, with adjusted odds ratios of 4.9 (95% CI 2.7–8.9) for men and 7.4 (95% CI 3.2–16.7) for women.

**Table 2:** Unadjusted and adjusted odd ratios (OR) predicting men’s and women’s likelihood of wanting no more children by HIV status.

HIV Status	Men (N = 1372)				Female (N = 1372)			
	Unadjusted		Adjusted		Unadjusted		Adjusted	
	OR	95 CI	OR	95 CI	OR	95 CI	OR	(95% CI)
Known Negative	1.0		1.0		1.0		1.0	
Known Positive	4.8	(2.9–8.1)***	4.9	(2.7–8.9)***	7.2	(3.6–14.5)***	7.4	(3.2–16.7)***

P< = 0.001*** (adjusted for own and partner education, woman and man experience of child mortality, partner’s age, woman total number of living children, men number of children from past marriage and woman experience of past marriage, marriage duration and knowledge of partner’s HIV status).

### Couples’ HIV Awareness and Fertility Intentions

Table 3 shows fertility intentions by the couples’ HIV status. The expressed desire for both to have no more children was highest 27/38 (71%) among concordant HIV positive couples and lowest 325/1260 (26%) among concordant HIV negative couples, with discordant couples having an intermediate value 33/61 (54%). Among concordant negative couples, husbands and wives were equally likely to want no more children. Among concordant positive and discordant couples, the majority agreed in wanting no more but, in cases of disagreement, it was the husband who was more likely to want no more.

**Table 3:** Couples’ fertility intention by knowledge of their HIV status.

Couples’ HIV status	Couples’ fertility intention outcomes							
	Both want more children/undecided		Both want no more children		Couple disagreements on intentions		Total	
	%	n	%	n	%	n	%	N
**Concordant Negative**	53	675	26	325	21	260	**100**	**1,260**
**Concordant Positive**	8	3	71	27	21	8	**100**	**38**
**Discordant Couples/don’t know**	16	10	54	33	29	18	**100**	**61**
Total	**50**	**688**	**28**	**385**	**21**	**286**	**100**	**1,359**

For the subsequent analysis couples with at least one member wanting more or undecided were combined with those where both wanted more or were undecided. Logistic regression in Table 4 shows that concordant HIV positive couples were more likely to desire to stop child bearing than were their negative counterparts: odds ratio 6.6 (95% CI 3.0–14.8), increasing to 11.2 (95%CI 4.7–26.9) after adjusting for possible confounders. We also found that discordant couples were also more likely to express the desire to stop child bearing compared to couples who reported being HIV negative or couples who reported that they did not know their HIV status.

**Table 4:** Unadjusted and adjusted odds ratios of couples’ fertility intentions by couples’ composite knowledge of their HIV status.

Couples HIV Status	Odds ratios (OR) of wanting no more children among monogamous couples			
	Unadjusted		Adjusted	
	OR	95% CI	OR	95 CI
Known Negative	1.0		1.0	
Concordant Positive	6.6***	3.0–14.8	11.2***	4.7–26.9
Discordant	3.1***	1.6–5.9	2.1	1.1–4.2

P< = 0.001*** (adjusted for own and partner education, woman and man experience of child mortality, own and partner’s age, woman total number of living children, men number of children from past marriage, type of dwelling and woman experience of past marriage, marriage duration and HIV status disclosure).

Among those who did not want more children the most important reason given varied. Among HIV negative women who wanted no more children, finances or effects on woman’s health were major foreseen adverse consequences, each reported by 291/351 (83%). Among HIV positive women effects on woman’s health was the major reason, reported by 15/20 (75%) of women on ART and 10/15 (67%) of women not on ART. For HIV negative men, 206/313 (66%) reported finances and 74/313 (24%) the woman’s health. Among HIV positive men on ART 14/33 (42%) reported women’s health and 9/33 (27%) child health. Among HIV positive men not on ART 6/18 (33%) reported woman’s health, 5/18 (28%) finances and 5/18 (28)% child health.

### Couples’ HIV Awareness and Contraception

Levels of contraceptive use reported by wives were almost identical among concordant negative, concordant positive and discordant couples (Table 5). Little difference was apparent between concordant couples in method choice, with hormonal methods and condoms equally popular. However, hormonal methods were less commonly used by discordant couples and condoms were more likely to be used. Contraception use did not seem to vary depending on whether it is the woman or the man who wanted more children. In unions where a woman wanted more children and the man wanted to stop 63/143 (44%) women used any modern method of contraception compared to 60/143 (42%) in unions where a woman wanted to stop and a man wanted more children. Logistic regression showed no significant difference in contraceptive use between couples by HIV status. Compared with concordant negative couples, adjusted odds ratio of use of any modern method of contraception in the concordant positive couples was 1.5 (95%CI 0.8–3.3). The odds ratio for discordant couples was 0.6 (95%CI 0.4–1.0) (Table 6). After controlling for HIV status and other background characteristics couples who agreed to stop child bearing were more likely to use any method of contraception (1.7 95%CI 1.2–2.4).

**Table 5:** Use of contraception by couples’ knowledge of HIV status.

CouplesKnowledge of HIV status	Contraception											
	Not using		Hormonal methods		Condoms		Permanent methods		Other methods		Total	
	%	n	%	n	%	n	%	n	%	n	%	N
**Concordant Negative**	52	674	19	257	18	242	7	85	4	44	**100**	**1,302**
**Concordant Positive**	48	14	21	6	17	5	14	4	0	0	**100**	**29**
**Discordant Husband positive**	50	13	4	1	38	10	8	2	0	0	**100**	**26**
**Discordant wife positive**	66	10	7	1	27	4	0	0	0	0	**100**	**15**
**Total**	**52**	**711**	**19**	**265**	**19**	**261**	**7**	**91**	**3**	**44**	**100**	**1,372**

**Table 6:** Logistic regression model presenting the probability of using contraception by couples’ awareness of their HIV status and stated intentions.

		Any contraceptive use	Odds ratio (95% CI) of using contraception	
		n/N (%)	Non-adjusted	Adjusted^1^
HIV status Knowledge				
Concordant negative (reference)	628/1302 (48)		1.0	1.0
Concordant Positive	19/39 (52)		1.0 (0.5–1.9)	1.5 (0.8–3.3)
Discordant/don’t know their status	23/63 (37)		0.6 (0.4–1.0)	0.7 (0.4–1.4)
Couple’s fertility intentions				
Both want more/undecided (Reference)	447/974 (46)		1.0	1.0
Both want no more	206/385 (54)		1.3 (1.0–1.7)	1.7 (1.2–2.4)

1 Adjusted for own and partner education, woman and man experience of child mortality, own and partner’s age, woman total number of living children, men number of children from past marriage, type of dwelling and woman experience of past marriage, marriage duration and HIV status disclosure). For couple’s fertility intentions also adjusted for HIV status knowledge.

## Discussion

In this population based cross-sectional study of monogamously married men and women we found that most HIV positive men and women wanted to stop child bearing. 39/56 (70%) of men on ART and 24/36 (69%) of men not on ART desired to stop child bearing. Similar proportions were found among women. These proportions are higher than those found in others studies [22], [23], [24]. Our findings confirm those of other individual level studies: that awareness of HIV positive status is associated with the desire to stop child bearing among married men and women [8], [25]. We do not see much effect of ART on couples’ fertility intentions. This could be because: 1) the ART program in this country was rolled out to rural areas three to four years later than the time ART program was initiated in urban areas [26] and therefore participants on the ART program have not been on the program long enough to fully appreciate the impact of the treatment on health; 2) high levels of stigma surrounding being HIV positive and ART enrolment could not only prevent people form accessing ART treatment but also prevent disclosure and open discussion in relation to sexual and reproductive health [27]; 3) the numbers of those on ART included in our analysis are too small for us detect the influence of ART on fertility intentions;. 4) HIV positive women and men on ART are counselled to avoid pregnancy so they may not want it and participants may have considered it socially desirable not to express desire for children when HIV positive. These expressed differences in fertility desires by couples’ awareness of their status could assist reproductive health services planners in designing programmes that address couples’ aspirations and needs especially when integrating family planning and HIV prevention interventions in the context of ART programmes.

Use of any modern method of contraception was slightly higher in this setting than in similar settings in Malawi [28]. 628/1302 (48%) of HIV concordant negative, 15/29 (52%) of HIV concordant positive, 13/26 (50%) discordant husband positive and 5/15 (44%) discordant wife positive used any method of contraception. Condom use, especially among HIV discordant couples, was lower than that reported in other studies [22]. Condoms have been described as ‘an intruder’ in marriage in rural Malawi and associated with extra-marital sex [29] and therefore couples may find it difficult to introduce condoms within marriage, especially if the HIV positive partner has not disclosed their status to their spouse [27].

There are a number of limitations of our study. Disclosure of HIV status in this setting and in similar settings has been associated with HIV negative results and therefore its influence on fertility intentions may have been on HIV negative couples more than on HIV positive couples or discordant couples [27]. Our analysis has focussed on monogamously married couples and therefore the findings cannot be generally applied to all couples including those in polygamous unions which constitute 20% of all married unions in the study population.

Due to small numbers our analysis has been unable to establish whether in discordant couples the effect of HIV status on fertility intentions differs depending on whether it is the husband or the wife who is positive. Our analysis however is one of the few studies on this subject involving relatively larger numbers of monogamously married couples.

### Conclusion

We have demonstrated that awareness of HIV positive status is associated with the desires to stop child bearing and that members of couples who both want no more children are more likely to use contraception – but contraceptive use is quite low. Greater promotion of an environment which facilitates condom use and contraceptive use within marriage in this and similar settings is warranted, such as couples counselling, linking HIV services and family planning, and taking into account couples’ expressed fertility preferences. As more HIV positive individuals and couples get on ART, we recommend a further analysis of the impact of ART on increased fertility desires and fertility. We also recommend interventions that promote couple disclosure of HIV status as this will assist couples in their efforts to fulfil their reproductive rights.

## References

[1] United Nations. Dept. of Economic and Social Affairs (2002) HIV/AIDS and fertility in sub-Saharan Africa: A Review of the research literature. Geneva: Population Division. 1–24.

[2] Zaba B, Gregson S (1998) Measuring the impact of HIV on fertility in Africa. AIDS 12 Suppl 1S41–50. 9677188

[3] Setel P (1995) The effects of HIV and AIDS on fertility in East and Central Africa. Health Transit Rev 5 Suppl: 179–189 10159889

[4] Magadi MA, Agwanda AO (2010) Investigating the association between HIV/AIDS and recent fertility patterns in Kenya. Soc Sci Med 71: 335–344. 20494502 10.1016/j.socscimed.2010.03.040

[5] Heys J, Kipp W, Jhangri GS, Alibhai A, Rubaale T (2009) Fertility desires and infection with the HIV: results from a survey in rural Uganda. AIDS 23 Suppl 1S37–45. 20081387 10.1097/01.aids.0000363776.76129.fd

[6] Taulo F, Berry M, Tsui A, Makanani B, Kafulafula G, et al (2009) Fertility intentions of HIV-1 infected and uninfected women in Malawi: a longitudinal study. AIDS Behav 13 Suppl 120–27. 19308718 10.1007/s10461-009-9547-9

[7] Hoffman IF, Martinson FE, Powers KA, Chilongozi DA, Msiska ED, et al (2008) The year-long effect of HIV-positive test results on pregnancy intentions, contraceptive use, and pregnancy incidence among Malawian women. J Acquir Immune Defic Syndr 47: 477–483. 18209677 10.1097/QAI.0b013e318165dc52

[8] Nduna M, Farlane L (2009) Women living with HIV in South Africa and their concerns about fertility. AIDS Behav 13 Suppl 162–65. 19301114 10.1007/s10461-009-9545-y

[9] Laher F, Todd CS, Stibich MA, Phofa R, Behane X, et al (2009) A qualitative assessment of decisions affecting contraceptive utilization and fertility intentions among HIV-positive women in Soweto, South Africa. AIDS Behav 13 Suppl 147–54. 19308719 10.1007/s10461-009-9544-z

[10] Cooper D, Harries J, Myer L, Orner P, Bracken H (2007) “Life is still going on”: reproductive intentions amongs HIV-positive women and men in South Africa. Social Science and Medicine 65: 274–283. 17451852 10.1016/j.socscimed.2007.03.019

[11] Yeatman S (2009) HIV Infection and Fertility Preferences in Rural Malawi. Studies in Family Planning 40: 261–274. 21151844 10.1111/j.1728-4465.2009.00210.x PMC2998897

[12] Yeatman SE (2009) The impact of HIV status and perceived status on fertility desires in rural Malawi. AIDS Behav 13 Suppl 112–19. 19301116 10.1007/s10461-009-9534-1 PMC3856651

[13] Maier M, Andia I, Emenyonu N, Guzman D, Kaida A, et al (2009) Antiretroviral therapy is associated with increased fertility desire, but not pregnancy or live birth, among HIV+ women in an early HIV treatment program in rural Uganda. AIDS Behav 13 Suppl 128–37. 18389364 10.1007/s10461-008-9371-7 PMC3606959

[14] Myer L, Morroni C, Rebe K (2007) Prevalence and determinants of fertility intentions of HIV-infected women and men receiving antiretroviral therapy in South Africa. AIDS Patient Care STDS 21: 278–285. 17461723 10.1089/apc.2006.0108

[15] Blanc A (2001) The effect of power in sexual relationship on sexual and reproductive health: an examination of the evidence Studies in Family Planning. 32: 182–213. 10.1111/j.1728-4465.2001.00189.x 11677692

[16] Becker S (1996) Couples and reproductive health: a review of couple studies. Studies in Family Planning 27: 291–306. 8986028

[17] Dodoo FN (1998) Men matter: additive and interactive gendered preferences and reproductive behavior in Kenya. Demography 35: 229–242. 9622784

[18] Bankole A, Singh S (1998) Couple’ fertility and contraception decision making in developing countries: hearing the man’s voice. International Family Planning Prospectives 24: 15–23.

[19] Jahn A, Crampin AC, Glynn JR, Mwinuka V, Mwaiyeghele E, et al (2007) Evaluation of a village-informant driven demographic surveillance system. Demographic Research 16: 219–248.

[20] Molesworth AM, Ndhlovu R, Banda E, Saul J, Ngwira B, et al (2010) High Accuracy of Home-Based Community Rapid HIV Testing in Rural Malawi. Journal of Acquired Immune Deficiency Syndrome 55: 625–630. 10.1097/QAI.0b013e3181f98628 PMC3248920 21934554

[21] Becker S, Sutradhar SC (2007) Fertility intentions: are the undecided more like those who want more or want no more children? J Biosoc Sci 39: 137–145. 16566846 10.1017/S0021932006001283

[22] Panozzo L, Battegay M, Friedl A, Vernazza PL (2003) High risk behaviour and fertility desires among heterosexual HIV-positive patients with a serodiscordant partner–two challenging issues. Swiss Med Wkly 133: 124–127. 12644959 10.4414/smw.2003.10124

[23] Loutfy MR, Hart TA, Mohammed SS, Su D, Ralph ED, et al (2009) Fertility desires and intentions of HIV-positive women of reproductive age in Ontario, Canada: a cross-sectional study. PLoS One 4: e7925. 19997556 10.1371/journal.pone.0007925 PMC2785467

[24] Cooper D, Moodley J, Zweigenthal V, Bekker LG, Shah I, et al (2009) Fertility intentions and reproductive health care needs of people living with HIV in Cape Town, South Africa: implications for integrating reproductive health and HIV care services. AIDS Behav 13 Suppl 138–46. 19343492 10.1007/s10461-009-9550-1

[25] Nattabi B, Li J, Thompson SC, Orach CG, Earnest J (2009) A systematic review of factors influencing fertility desires and intentions among people living with HIV/AIDS: implications for policy and service delivery. AIDS Behav 13: 949–968. 19330443 10.1007/s10461-009-9537-y

[26] Floyd S, Molesworth A, Dube A, Banda E, Jahn A, et al (2010) Population-Level Reduction in Adult Mortality after Extension of Free Anti-Retroviral Therapy Provition into Rural Areas in Malawi. Plos One 10: e13499. 10.1371/journal.pone.0013499 PMC2957442 20976068

[27] Anglewicz P, Chintsanya J (2011) Disclosure of HIV status between spouses in rural Malawi. AIDS Care 23: 998–1005. 21390889 10.1080/09540121.2010.542130 PMC3371657

[28] National Statistical Office (NSO)[Malawi] aOM (2010) Malawi Demographic and Health Survey 2010. Calverton, Maryland: NSO and ORC Macro.

[29] Chimbiri A (2007) The condom is an ‘intruder’ in marriage: Evidence from rural Malawi Science and Medicine. 5: 1102–1115. 10.1016/j.socscimed.2006.10.012 17240504

